# The effect size, study design, and development experience in commercially sponsored studies for new drug applications in approved drugs

**DOI:** 10.1186/2193-1801-3-740

**Published:** 2014-12-15

**Authors:** Satoshi Fukunaga, Makiko Kusama, Shunsuke Ono

**Affiliations:** Laboratory of Pharmaceutical Regulatory Science, Graduate School of Pharmaceutical Sciences, The University of Tokyo, 7-3-1 Hongo, Bunkyo-ku, Tokyo, 113-0033 Japan

**Keywords:** Study design, Development experience, New drug application, Randomized controlled trial, Effect size

## Abstract

Pharmaceutical companies incorporate different features into the trials for new drug applications (NDAs) to render them efficient, making use of their experience. The objective of this analysis was to examine the associations between outcome and features related to study design and clinical development experience in commercially sponsored clinical trials.

We collected data of phase 2 and phase 3 trials of all the drugs that obtained approval for depression, schizophrenia, asthma, hypertension, and diabetes in Japan from 1970 to 2011. In total, 145 trials from 90 test drugs were eligible for our study. We calculated the effect size, the standard mean of differences between test drug and comparator therapeutic effects, as the objective variable for use in our analysis. A linear mixed effect model with nested and crossed random effects was used in the analysis including variety of therapeutic area, test drugs and clinical trials.

The analysis showed that trial features including sample size, subjective endpoints and active comparator of the same mode of action were negatively associated with effect size. In addition, sponsors’ domestic clinical development experience with similar drugs seemed to have a positive association, but prior development experience in foreign countries did not. The accumulation of skills and knowledge within sponsors, and accumulated experience in domestic professionals who implement clinical trials under study contracts with sponsors would be of great importance for yielding clear outcomes.

This study provides additional evidence with respect to possible sizes and directions of the influence of study design features that must be considered when planning and implementing trials for new drug applications, and when retrospectively comparing outcomes from trials with different designs and environments.

**Electronic supplementary material:**

The online version of this article (doi:10.1186/2193-1801-3-740) contains supplementary material, which is available to authorized users.

## Background

It has been suggested that recent stagnation in drug development is related to both a decrease in the probability of success, and an increase in the costs of research and development (Pammolli et al.^2011^). This has led pharmaceutical companies and even regulatory agencies to address urgent priorities, in an effort to halt this decline in the rates of successful clinical development.

Conducting well-designed clinical trials and obtaining positive results are necessary for the approval of a new drug. In commercially sponsored clinical trials, it is pharmaceutical companies that make decisions on key components of study design, to achieve their goals most efficiently within their practical constraints. Previous studies have indicated that clinical trial outcomes can be affected by design components such as the number of treatment arms, sample size, the probability of receiving a placebo, year of publication, baseline severity of disease, and dose regimen (Khan et al.^2004^; Papakostas and Fava^2009^; Kirsch et al.^2008^). These studies mainly targeted clinical trials in psychiatry with subjective efficacy endpoints; trials with objective endpoints in other therapeutic fields have not been reviewed intensively.

Companies' development-strategy oriented choices and behaviors may also have a substantial impact on study outcomes (Lundh et al.^2012^). Pharmaceutical companies are usually interested in generating evidence to support clinical efficacy and safety at the minimum cost and burden, which often depends on their experience, and their choice of strategy. In this global development era, global companies can choose the region in which they initiate clinical development and submit NDAs first, after assessing the likelihood of success, costs, and future profits. Preceding drug development experience in one region will contribute to subsequent development in other regions. In addition to global development pathways, domestic development experience with regard to similar drugs seems to affect the design and conduct of clinical trials, through the experience of companies as well as investigators and institutions. However, previous studies have not sufficiently investigated such aspects of experience.

The aim of the present study was to analyze in what way study design features and development experience were associated with the effect size of trial outcomes in both placebo and active-controlled clinical trials for depression, schizophrenia, hypertension, asthma, and diabetes drugs. Many of the previous studies were focused on specific therapeutic areas, anti-depressant trials in particular (Khan et al.^2004^; Papakostas and Fava^2009^; Kirsch et al.^2008^; Rutherford et al.^2009^; Rief et al.^2009^). Samples of clinical trials targeted in previous research were collected mainly from the international clinical trial databases, and were composed of both commercial and investigator-initiated trials. In this study, we aimed at commercial trials that were part of the NDA clinical data package in Japan, and investigated possible associations between the trial outcome and relevant factors. This made it possible for us to establish and test research hypotheses on drug companies’ behaviors, based on data from various phases of clinical trials for different statistical purposes. Associations between clinical trials with common attributes (*e.g*., trials for the same test drug in different phases) were assessed via a nested random variable structure as explained in the Methods section below. We analyzed several types of endpoints and clinical trials, including physical endpoints and active-controlled trials that few previous studies have analyzed.

## Methods

### Source data

We collected clinical trial data from phase 2 and phase 3 randomized double-blind comparative trials of all the drugs that obtained approval for depression, schizophrenia, asthma, hypertension, and diabetes as new molecular entities or new indications in Japan from 1970 to April 2011. All pivotal phase 3 trials and phase 2 trials that determined final dose and regimen were included regardless of their outcomes. For some drugs including escitalopram and ketotifen, clinical trials that did not achieve pre-determined objectives were included in the database. For the NDA of duloxetine the first NDAs were rejected by the authority because of failure to establish efficacy, and the second NDAs were submitted with additional confirmatory trials. We chose these therapeutic fields in order to evaluate the possible impact of endpoint choices, some of which were objective and others subjective, phases of clinical trials, statistical purposes such as examining superiority, non-inferiority and equivalence, types of comparators (placebo or active comparator), and the use of clinical global impression of improvement (CGI-I).

Data were extracted from common technical documents posted on the Japanese regulatory agency (the Pharmaceutical and Medical Devices Agency) site (http://www.info.pmda.go.jp/), from the interview forms, which are unique to Japan, from pharmaceutical company’s websites, and from publications on clinical trials conducted for NDAs (Additional file1: Table S1). We determined a pairing of the test drug and a comparator for each trial. In the case of three-armed trials with a test drug, active control, and placebo, we assessed two pairs; *i.e*., the test drug and the active control, and the test drug and the placebo and adjusted their possible associations in the nested random-effect model.

### Effect size

Differences between test drug and comparator therapeutic effects were calculated using Hedges' adjusted g for continuous endpoints. Therapeutic effect was defined as the overall change from baseline to final assessment. Standard deviation values were not reported in 2 of 145 clinical trials. The values were imputed from the standard deviation of the other trials with the same endpoint by using the mean imputation method (Leucht et al.^2009^). The sign and size of coefficients in a multi-level regression model was robust even if we removed the data of these two clinical trials from our dataset. CGI-I scale scores that corresponded to moderate clinical improvement or greater at the final visit were regarded as effective, and others were regarded as ineffective. We applied phi coefficients for CGI-I as effect sizes. For trials with more than one test drug dose arm, we selected dose arms within a range of approved standard maintenance daily dose, and calculated the mean effect size of those dose arm comparator pairs. Approved maintenance daily dose was defined as approved dose excluding the dose only used in titration period.

Effect sizes are under influence of many factors and interactions thereof in both planning and conducting stages, including agreement with regulators, efficacy of a drug *per se*, alpha and beta risks (*i.e*., power) set at the planning stage, targeted label claims, types of endpoints, and quality of conduct. The effect size also satisfies the following statistical equation by definition;

$$T=f\left(N\right)*g\left(ES\right)$$

where T is the test statistic, N is the sample size, ES is the effect size, and f (.) and g (.) are some function forms. It should be noted that if there is some constraint on T (e.g., pre-determined levels of alpha and beta risks), as is in most late phase trials, ES and N could show spurious negative association reflecting this statistical relationship. The purpose of this analysis was to explore possible associations between the effect size and several observable attributes of clinical trials and provide additional clues to historical discussions on how critical study design components and company attributes could affect study outcomes.

### Statistical analysis

The differences in effect size across diseases were tested using the Kruskal-Wallis one-way analysis of variance by ranks and two-sample t test with equal variances.

We constructed a multi-level regression model with effect size as the objective variable, whose nested structure was basically similar to those of previous studies (Hedeker et al.^1994^). A linear mixed effect model with nested and crossed random effects was used in this analysis. We expanded a construction of random effects in previous studies (Rutherford et al.^2009^; Woods et al.^2005^), as it seemed reasonable to assume that effect sizes of test drugs within the disease were correlated and that effect sizes of trials within a test drug were also correlated. Diseases, test drugs, and trials were treated as hierarchical random effects, which enabled to accommodate the nested structure of subjects within trials within test drugs clustered by diseases. We treated comparators as a crossed random effect with test drugs because there were multiple pairs of a test drug and several comparators, and vice versa. The form of the model was as follows:

$$\begin{array}{ll} Y_{\mathrm{ijkl}}= & \beta_{0}+\beta_{1}x_{1jkl}+\ldots +\beta_{\mathrm{ijkl}}x_{\mathrm{ijkl}}+\zeta_{i}+\zeta_{ij}+\zeta_{ijk}\\ +\zeta_{l}+{ɛ}_{\mathrm{ijkl}} \end{array}$$

Y_ijkl_: effect size, x_ijkl_: covariates, ζ: random effects, i: disease, j: test drug, k: trial, l: comparator.

We adopted four regression models: Models 1 and 3 with explanatory variables related to study design, and Models 2 and 4 with clinical development variables (*i.e*., precedent foreign clinical trial data and companies’ domestic development experience with similar drugs) in addition to the variables in Model 1 or 3. Models 3 and 4 were for subgroup analyses with the dataset including only phase 3 trials, using the same regression model as Models 1 and 2 except the phase 2 dummy variable.

We classified a company as having had (or shared, at least) precedent development experience in foreign countries in cases where the company submitted foreign clinical trials in the Japanese NDA. A company was classified as having had domestic development experience with similar drugs in cases where it had gained at least one drug approval for the same therapeutic indication in Japan before the approval of the drug we analyzed. The significance level was set at p < 0.1 as in previous studies (Hirai et al.^2012^). The analysis was performed using Stata12 (StataCorp, College Station, TX).

## Results

### Distribution of effect size

**Figure 1:**
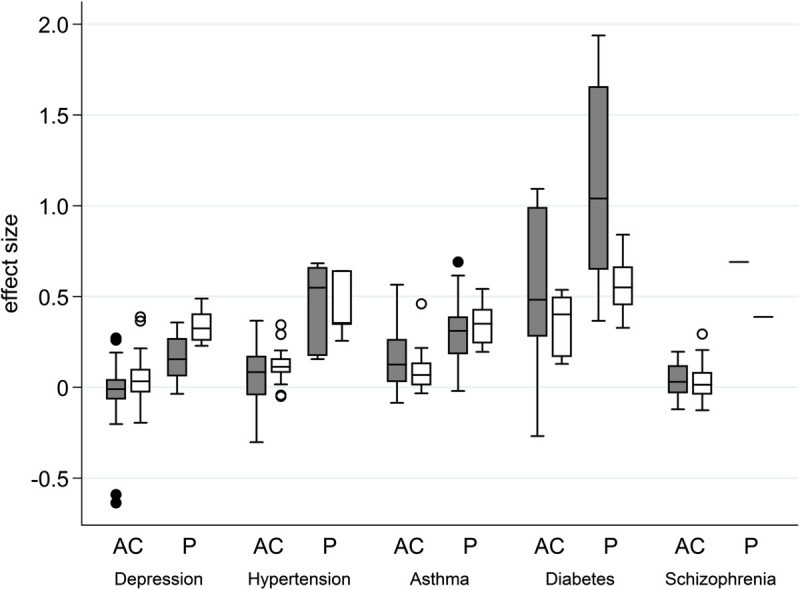
**Box**-**whisker plot of effect sizes of each disease.** Gray box: observed effect sizes, white box: predicted effect sizes, AC: effect size of the pairing of a test drug and an active comparator, P: effect size of the pairing of a test drug and placebo. The boxes show interquartile ranges. The horizontal line across each box denotes the median, and vertical lines extending above and below each box indicate the minimum and maximum values. Dots above and below the boxes are outliers.

In total, 145 trials from 90 test drugs were eligible for inclusion in our study. Descriptive data relating to the collected variables are shown in Table 1. The 145 trials consisted of 33 depression, 25 schizophrenia, 27 asthma, 38 hypertension, and 22 diabetes trials. There were 44 pairs of a test drug and a placebo, and 150 pairs of a test drug and an active comparator. Effect sizes ranged from -0.64 to 1.94, and the average was 0.19. Fifty one of 55 negative effect sizes were observed in active-controlled trials. Other 4 negative effect size were observed in placebo-controlled trials for depression (duloxetine and escitalopram) and for bronchial asthma (ketotifen and tranilast), although the trial of tranilast was not considered to be failed because a favorable result was shown in the primary endpoint, CGI-I. The mean effect sizes of failed and successful trials in the NDAs of duloxetine, escitalopram and ketotifen were -0.020 and 0.164, respectively. Table 1 summarizes the characteristics of collected clinical trials. A box-whisker plot of effect size by disease is shown in Figure 1. Effect sizes were significantly different across diseases (a test drug-placebo pair: p = 0.0001, a test drug-active comparator pair: p = 0.0001). Further, we found a statistically significant difference in the means of effect size between diabetes drugs and non-diabetes drugs (p < 0.0001).

**Table 1:** **Summary statistics of explanatory variables and effect sizes**

Variable			n	%
Total number of test drug-comparator pairs observed			194	100
Endpoint	HDRS		19	10
	PANSS		12	6
	PEF		16	8
	Blood pressure		41	21
	HbA1c		24	12
	CGI-I	Depression	33	17
		Schizophrenia	25	13
		Asthma	24	12
Comparator	Placebo		44	23
	Active		150	77
Statistical purpose	Superiority		32	17
	Non-inferiority and equivalence		57	29
	Dose response		20	10
	Other		85	44
Number of arms	2		142	73
	3		28	14
	4		22	11
	5		2	1
	All		194	2.40 (0.729)^a^
Sample size			194	236 (108)^a^
Length of trial (weeks)			194	8.54 (4.10)^a^
Mean number of subjects per site			182	5.14 (3.20)^a^
Approval year			194	1999^b^
Dosing schedule	Flexible		109	56
	Fixed		85	44
Type of endpoint	Subjective endpoint (assessment score)		30	15
	Subjective endpoint		84	43
	(Clinical Global Improvement)			
	Objective endpoint		80	41
Primary endpoint				
	Yes		147	76
	No		47	24
Comparator with the same mode of action of the test drug				
	Yes		153	79
	No		41	21
Phase2				
	Yes		36	19
	No		158	81
Precedent foreign clinical trial data				
	Yes		83	43
	No		111	57
Companies' domestic development experience with similar drugs				
	Yes		91	47
	No		103	53
Mean age of subjects			193	48.0 (7.63)^a^
Ratio of female subjects			194	0.47 (0.10)^a^
Effect size	Placebo		44	0.58 (0.52)^a^
	Active		150	0.08 (0.22)^a^

^a^Mean (SD), ^b^Median.

Precedent foreign clinical data: a dummy variable that was assigned a value of 1 if foreign clinical trials were included in the clinical data package. Companies’ domestic development experience with similar drugs: a dummy variable that was assigned a value of 1 when a company had gained at least one drug approval for the same therapeutic indication in Japan before the approval of a drug we analyzed.

HDRS: the Hamilton Depression Rating Scale for depression, PNASS: Positive and Negative Syndrome Scale for schizophrenia, PEF: morning Peak Expiratory Flow for asthma, Blood pressure: diastolic pressure for hypertension, HbA1c: hemoglobin A1c for diabetes, CGI-I: Clinical Global Impression of Improvement.

The mean effect size in diabetes was much larger than those in other indications (diabetes: 0.48, other indications: 0.04-0.17). Larger mean effect sizes in diabetes were observed clearly even in the pairs with active-control drugs (diabetes: 0.35, other indications: 0.03-0.12). Negative effect sizes were observed for some trials, which reflected the fact that there were both successful and failed trials included in the data packages of successfully approved drugs.

### Regression analysis

The results of the regression analysis are summarized in Table 2. The analysis indicated that approval year, superiority trials, and primary endpoints were positively associated with effect sizes (Model 1). Sample size, subjective endpoint evaluated with assessment scores (the Hamilton Depression Rating Scale for depression, Positive and Negative Syndrome Scale for schizophrenia), subjective endpoint evaluated with CGI-I, the same mode of action of the study drug and the active comparator, and the proportion of female subjects were negatively associated with effect size (Model 1). Among trials with different statistical purposes, superiority trials were positively correlated with effect size (b = 0.245, 90% confidence interval 0.115-0.375, p = 0.002). This result was predictable because most of the superiority trials were placebo-controlled trials and our dataset was composed of trials for NDAs that were successfully approved.

**Table 2:** **Mixed effect regression analysis of effect sizes**

		Model 1 (N = 182)		Model 2 (N = 182)		Model 3 (N = 149)		Model 4 (N = 149)	
		Estimate	***P*** value	Estimate	***P*** value	Estimate	***P*** value	Estimate	***P*** value
Number of arms		0.070	0.102	0.055	0.208	0.073	0.282	0.077	0.241
Sample size		-0.001	0.007***	-0.001	0.008***	-0.001	0.002***	-0.001	0.000***
Length of trial		0.004	0.495	0.002	0.697	0.003	0.593	0.000	0.957
Flexible dosing schedule		-0.072	0.190	-0.070	0.205	-0.098	0.062*	-0.112	0.030**
Approval year		0.007	0.028**	0.004	0.307	0.005	0.166	0.000	0.979
Mean number of subjects per site		0.009	0.147	0.007	0.219	0.011	0.065*	0.008	0.171
Type of endpoint (Objective endpoint^a^)	Subjective endpoint (assessment score)	-0.138	0.022**	-0.119	0.048**	-0.090	0.136	-0.067	0.263
	Subjective endpoint (Clinical Global Improvement)	-0.115	0.033**	-0.094	0.081*	-0.076	0.190	-0.049	0.395
Statistical purpose (Others^a^)	Superiority	0.245	0.002***	0.215	0.009***	0.287	0.000***	0.229	0.005***
	Noninferiority and equivalence	-0.005	0.933	-0.028	0.700	0.012	0.853	-0.055	0.447
	Dose response	0.133	0.269	0.124	0.304	-	-	-	-
Primary endpoint		0.095	0.018**	0.086	0.032**	0.061	0.152	0.050	0.234
Comparator with the same mode of action of the test drug		-0.127	0.018**	-0.143	0.006***	-0.083	0.124	-0.129	0.020**
Phase2		-0.067	0.384	-0.059	0.447	-	-	-	-
Mean age of subjects		0.006	0.142	0.008	0.075*	0.013	0.004***	0.015	0.001***
Proportion of female subjects		-0.417	0.053*	-0.453	0.037**	-0.752	0.002***	-0.682	0.004***
Precedent foreign clinical trial data		-	-	0.073	0.329	-	-	0.167	0.029**
Companies‘ domestic development experience with similar drugs		-	-	0.079	0.078*	-	-	0.086	0.051*
Constant		-0.148	0.565	-0.098	0.706	-0.200	0.510	-0.168	0.572

^a^Reference category, *p < 0.1, **p < 0.05, ***p < 0.01.

Companies’ domestic development experience in Japan with similar drugs was positively associated with effect size in Model 2 (b = 0.079, 90% confidence interval 0.005-0.153, p = 0.078), but previous experience of foreign development was not. We conducted subgroup analysis of only phase 3 trials using the same regression model except phase 2 dummy variable. The results are shown as Models 3 and 4 in Table 2. Precedent foreign experience as well as companies’ domestic development experience with similar drugs was positively associated with effect size in phase 3 trials (b = 0.167; 90% confidence interval 0.041-0.293, p = 0.029).

The random effects variance estimates for the mixed effect model (Model 1) are 0.019 for diseases, 0.008 for test drugs, 0.014 for trials, and 0.018 for residuals. These random effects accounted for 69% of the total residual variance. As the total residual variance was 0.106 for a null model (a model without covariates) and 0.059 for the model including all covariates, 44% of the variance was explained by the covariates.

We conducted post-estimation of effect size after the regression in Model 1. Predicted effect sizes are shown in Figure 1. The current hierarchical regression model seems to be useful in predicting effect sizes in general, but there are still significant variations left unexplained by the model. Figure 1 also shows that it varies depending on drug class to what extent our model can predict effect sizes in the real world.

## Discussion

Thorough planning and implementation of clinical trials is the key to demonstrating a test drug's therapeutic potential and to meeting regulatory requirements for new drug approval. Success of clinical development undoubtedly depends on the quality of the study design and implementation, and pharmaceutical companies choose critical components of design and execution aimed at achieving the highest possible levels of success within the relevant budgetary and technical constraints. In this research, we investigated possible links between components of the study design and the effect size (*i.e*., the standardized outcome of various clinical trials) and found significant associations between them. We also investigated whether study environment and the prior experience of drug companies were associated with effect size, and some variables yielded statistically significant associations.

**Figure 2:**
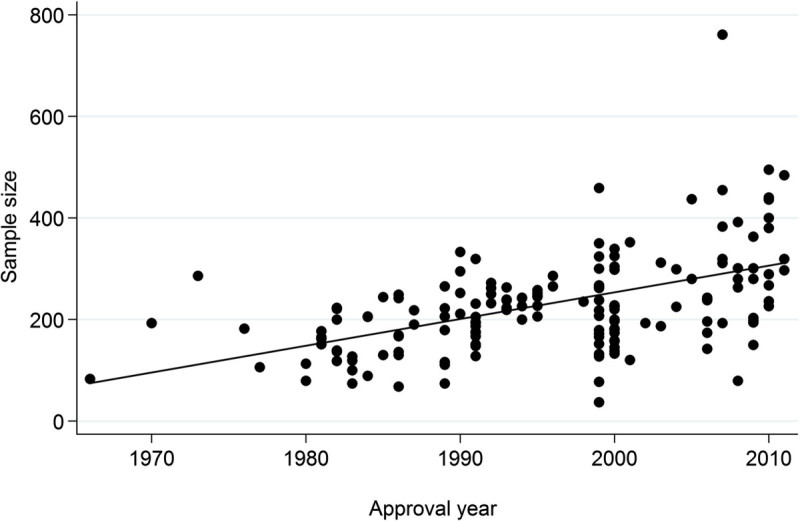
**Changes in the sample size by approval year.**

Among the variables related to study design, a negative correlation was found between the sample size and the effect size. No consistent correlations have been reported in other studies (Khan et al.^2004^; Papakostas and Fava^2009^; Yildiz et al.2011a), and no persuasive explanation was apparent for these associations. One plausible explanation for our result is that in our research targeting only approved drugs, most drug companies successfully achieved *p* values of approximately the same level (*i.e*., close to 0.05 or less) in clinical trials, which could lead to an inverse relationship between the effect size and the sample size. This is likely to reflect the statistical equation, T = f (N) * g (ES) (see Methods), but it is difficult in this model to distinguish this spurious association from substantial (*i.e*., causal) relationship of interest, if any. It is interesting to note that the negative association was observed even when recent increases in sample size were controlled by the time-trend variable “approval year” in Model 1. As Figure 2 shows, the sample size was larger in more recent trials in our dataset (r = 0.49), and similar trends have been observed in trials submitted to the US Food and Drug Administration (Khin et al.^2011^).

The number of arms, another component of study design, was not associated with effect sizes. It was previously reported that more number of arms resulted in greater effect sizes in placebo-controled anti-depressant trials (Khan et al.^2004^). In past decades, two-arm confirmatory trials with an active-comparator were common in Japan (Ono et al.^2002^). Since the introduction of the International Conference on Harmonisation (ICH) E10 guideline (The International Conference on Harmonization^2000^) and recent clinical evaluation guidelines for each therapeutic field that require a concurrent placebo control group, however, comparative trials with both concurrent positive comparators and a placebo arm, are expected to increase. Our results do not necessarily reflect these changes after the ICH E10, and caution is required when extrapolating them.

The time-trend variable, approval year, has various implications, including changes in patient populations and background therapies, and it seems likely that various mechanisms influenced the observed correlation. Stricter inclusion and exclusion criteria in recent trials to improve homogeneity may yield more focused outcomes (Rief et al.^2009^). Larger effect sizes in recent trials might also reflect drug companies' general preference to develop more effective drug candidates than existing drugs in response to stricter requests from the regulatory agency and healthcare professionals and to get ahead of strong market competition.

Using an active comparator with the same mode of action of the test drug seemed to yield smaller effect sizes, with statistical purposes controlled. In such trials the test drugs are generally less novel and innovative, thus this result seems logical.

Regarding endpoints, clinical trials using subjective endpoints showed smaller effect sizes than those using objective endpoints, although we cannot deny the possibility that the negative coefficient for CGI-I, a categorical variable, reflects some heterogeneity introduced by the difference in conversion to effect sizes. In this analysis we defined “subjective endpoints” as those evaluated via clinicians' subjective interpretations of patients' responses. A substantial difference between observer-rating scales and self-reporting scales has been reported in some therapeutic fields (Rief et al.^2009^; Bullens et al.^2001^). This may make it difficult to detect modest test drug/comparator differences in patients' symptoms.

Primary endpoints predicted higher effect sizes than secondary endpoints. Since our dataset consisted of successfully approved NDAs, endpoints tagged as “primary” were generally expected to be more efficient than secondary endpoints.

The proportion of female subjects was negatively related to effect size. It is reported that women and men can respond differently to drug treatments, particularly psychological agents (Khan et al.^2004^). Some previous reports have suggested positive associations between the male proportion and study outcomes (Khan et al.^2004^; Yildiz et al.2011b), which is in line with our observation. It is still difficult to predict, however, that increasing proportions of male subjects may yield better outcomes, because the gender proportion in clinical trials seems confounded by several factors affecting both the gender proportion and clinical outcomes, and it is almost impossible to adjust such potential confounders in retrospective analysis. Although drug companies routinely examine possible differences in efficacy by sex in study reports, they rarely become a focal point of discussion in the publications such as common technical documents for NDAs and review reports.

We were also interested in the possible influences of sponsors (*i.e*., drug companies), and the environment of clinical development of the drug, on effect sizes. Companies can use their previous data and experience, which vary greatly between companies, when designing and conducting trials. Regarding study environment, a previous analysis has shown that the development lag between Japan and the US was positively associated with the probability of transition from Phase 2 to Phase 3 trial and from Phase 3 trial to registration (Hirai et al.^2012^). It has been reported that the accumulation of experience also positively affects the success rate of clinical development (Danzon et al.^2005^). We included two explanatory dummy variables in Model 2 and Model 4, "Precedent foreign clinical trial data" and "Companies’ domestic development experience with similar drugs." The former incorporated the foreign clinical development experience regarding the drug prior to Japanese clinical development. The latter was included to explore possible roles of prior development experience of drugs in the same therapeutic class in Japan. We considered only successful experience (*i.e*., experience of NDAs approved) in this study as the explanatory variable due to practical difficulties in defining experience of a drug company and obtaining reliable data of unsuccsessful development projects. It should be noted, however, that both successes and failures actually consist of a company's development experience.

The results of Model 2 in Table 2 including both phase 2 and 3 trials show that companies' domestic development experience was positively associated with effect size, but use of foreign clinical trial data was not. In the subgroup analysis aiming at only phase 3 trials (Model 4 in Table 2), however, use of foreign clinical data as well as domestic experience had positive impact on effect size. They suggested that domestic development experience might lead to the accumulation of skills and knowledge within drug companies. Improvement in study design, for example, would be the key to establishing unequivocal results. Another possibility might be ascribed to accumulated experience in domestic clinical trial professionals with whom companies make clinical study contracts. Appropriate planning, design, and conduct of clinical trials largely depends on the skills of such professionals, and their skills could be improved by previous experience. Our results may support the latter possibility in that precedent development experience in foreign countries did not necessarily result in enhanced effect sizes in early exploratory phases. Difficulties in extrapolating foreign clinical data and experience to the Japanese environment, probably due to the differences of intrinsic and extrinsic factors such as medical practice and therapeutic approach, might also confound the situation. In phase 3 confirmatory trials, drug companies can make the most of all the preceding evidence in both Japan and other countries. Even in cases where ethnic differences have substantial impact on clinical development, companies at this last stage of development might be able to cope with such differences, optimizing planning, design, and conduct of Japanese phase 3 trials, and successfully achieve larger effect sizes based on previous domestic and foreign experience.

Our study suggests that we need to be cautious about trial design features and also drug companies’ experience when comparing the results of clinical trials. There were examples in which those features could have played some role in explaining differences in trial outcomes. Re-evaluation of a drug class termed “cerebral circulation and metabolism improver” in Japan during the 1990s was a historical case. Four out of five drugs in this class were withdrawn in 1998 because they failed to establish superiority to placebo in clinical trials for the re-evaluation ordered by the Ministry of Health and Welfare (MHW) (Hayashi et al.^1998^; The Ministry of Health and Welfare.^1998^). All of them had been approved based on equivalence studies in comparison with calcium hopantenate. The MHW justified its initial decisions of approval in the late 1980s and ascribed the results to changes in the healthcare environment such as advances in early diagnosis, surgical procedures, basic care, and rehabilitation. In addition to those healthcare environment changes, differences in study design between trials for initial approval and those for re-evaluation were noted.

### Limitations

Several limitations in our study need to be considered. First, our research focused on trials that were conducted in Japan and submitted for Japanese NDAs. We need to be cautious in extrapolating our current results to different regions. The homogeneity of the study populations of the trials has advantages, however, because it enables us to exclude possible confounders related to heterogeneity of race and ethnicity, while maintaining sufficient variety with regard to study design and developmental phase. Second, clinical trials submitted for NDAs are somewhat restricted in design and quality, compared to trials that are not conducted specifically for NDAs. Third, it should also be noted that the trials included in this study mostly constituted successful research, in that they were chosen on the basis of approval decisions. Fourth, the number of placebo-controlled trials investigating drugs for psychological disorders was small in our dataset because only a small number of trials of that kind were conducted in Japan during the observation period. Fifth, although we know agreements between the regulatory agency and the pharmaceutical companies could have significant impact on study design and outcomes, we were not able to collect data on meetings and agreements due to difficulties in accessing in-house development histories.

## Conclusions

In conclusion, this study showed significant associations between outcomes, study design features, and companies’ experience. To further advance this research, it is necessary to expand our focus and include unsuccessful trials and unapproved drugs. Recent trends toward registry and disclosure of clinical trial results would be helpful for this purpose (International Federation of Pharmaceutical Manufacturers and Associations.^2009^,^2010^). Further investigation is also needed to determine why these associations are apparent in clinical trials. Approaches based on psychology and cognitive sciences may be useful to clarify the influence of clinical investigators’ and patients’ responses to specific incentives and/or situations. Our results, in conjunction with clarification of the mechanisms behind the observed associations, could contribute to improving the quality and efficiency of clinical trials.

## Electronic supplementary material

Additional file 1: Table S1: List of data source. (XLS 83 KB)

## Abbreviations

NDA: New drug application
CGI-I: Clinical global impression of improvement
ICH: The International Conference on Harmonisation
MHW: The Ministry of Health and Welfare.
